# Analysis of health service utilization of migrants in Beijing using Anderson health service utilization model

**DOI:** 10.1186/s12913-018-3271-y

**Published:** 2018-06-18

**Authors:** Shuang Shao, Meirong Wang, Guanghui Jin, Yali Zhao, Xiaoqin Lu, Juan Du

**Affiliations:** The School of General Practice and Continuing Education, Capital Medical University, Beijing, 100069 China

**Keywords:** Migrants, Health seeking behavior, Anderson health service utilization model, Influence factors

## Abstract

**Background:**

Migrants are the unique production of China’s urbanization process. They are often excluded from social welfare and security systems of cities, and often exposed to high health risk related closely to their health problems. This research sought to unveil and explore the influencing factors on health services utilization of migrants in Beijing.

**Methods:**

A sample of 2014 inter-provincial migrants and 4578 residents with Beijing “*Hukou*” who were 15 years old and above was chosen by three-stage stratified cluster sampling method. A structured questionnaire survey was conducted via face-to-face interviews. Anderson health service utilization model was used to demonstrate the effects of the explanatory variables on health seeking behavior from predisposing, enabling and need variables.

**Results:**

The study reveals that the rate of ‘having symptoms’ of migrants was lower than that of residents with “*Hukou*” only in the group of 25 to 34 years old in the past month. 503 migrants (25.0%) and 1441 (31.5%) residents with “*Hukou*” reported at least one episode of discomfort in the past month, and the rate of health service seeking behavior among migrants (46.8%) was lower than residents with “*Hukou*” (62.6%) (*P* < 0.0001). Chi-square independence test shows that age, ethnicity, employment status, having chronic disease and the degree of symptom were the major determinants affecting migrants to receive health services. The binary logistic regression indicates that the degree of symptom as the need variable and ethnicity as the predisposing variable were the strong and consistent determinants of health services seeking behavior. The migrants with moderate degree and severe degree of symptom in the past month were at 1.623-times (OR = 1.623) and 5.035-times (OR = 5.035) higher chances of seeking health services respectively, comparing to mild degree of symptom. Minority migrants were less likely to seek health services than Han migrants (OR = 0.282).

**Conclusions:**

The results indicate that the current health delivery system is not conducive for migrants to seek appropriate health services. Relevant policies and feasible measures, including increasing the coverage of health insurance and improving the health perception of migrants should be vigorously implemented to provide affordable health services and change health service utilization behaviors for migrants.

## Background

China has a highly mobile inter-province or rural-to-urban population in the past and coming decades. The estimate of migrants increased from 230 million in 2011 to 247 million in 2015, which constituted 18% of the total population of China [1, 2]. As the political, economic and cultural center of China, Beijing attracts a large number of migrants from all over the country every year. In 2010, the sixth national census data showed that there were 7.045 million migrants in Beijing, and the proportion of migrants increased from 18.9% in 2000 to 35.9% in 2010 among the whole population in Beijing [3, 4]. In the process of migration flows, migrants contribute significantly to urban socioeconomic development and social stability. While, they are notoriously marginalized in China, especially due to a rigid household registration system-called “*Hu*kou”, that serves as a domestic passport, which regulates population distribution and rural-to-urban migration. Under the “*Hukou*” system, migrants cannot share most of the privileges as urban dwellers do, e.g. health insurance in local city. Moreover, migrants also experience life transitions, such as physical environment, lifestyle, cultural milieu modifications, occupational and socioeconomic changes [5–7]. Most of them were likely to be young, poor, less educated, and they generally tended to subsist in unstable, poorly sanitized and overcrowded living arrangements, and insecure employment conditions [8, 9]. In the unstable context with a lack of family supports, public infrastructure and social support network, migrants suffer from unnoticed high health risks that can wear off their health risk awareness and make them vulnerable to long-term health problems [10–12].

Basic health services in China have been recognized as one of the “essential rights” for everyone. Given the size of migratory population and their roles in accelerating the modernization and industrialization process, the health status and access to health behaviors of migrants have become a significant public health problem in China. It is undeniable that there are several barriers for migrants to seek health services because of the temporary and informal nature of their employment. Lack of insurance coverage, high cost of healthcare, insufficient knowledge and negative attitude to the health preventive behaviors, and demanding work schedules have resulted in migrants’ use of unsupervised self-treatment or substandard care [13–16]. Inadequate health services utilization put them at risk for poorer health outcomes than the local-born populations (e. g. high prevalence of environmental diseases, infectious diseases, and stress disorders) [17, 18]. Furthermore, the health status and health seeking behaviors of migration have impacts on the health care delivery system too, which exacerbates the burdens on medical resources and health serivces management system in immigrant cites.

In this regard, more attentions should be paid to the health risk and health-seeking behavior of these migrants, which is a vital public health challenge faced by government and society. Studying health and health seeking behavior in the context of migration offers a better understanding of the complexity and diversity of the migration process which is critical, as migration has become a widespread and persistent phenomenon that is changing the structure of family units, communities and societies in our modern world.

Systematic research on health services seeking behavior of Chinese migrants in illness status is far from sufficient. It is crucial to prevent disease, provide health promotion measures for the migrants in China. This research represents one of the first attempts to examine the potential major determinants of health seeking behaviors for migrants by using the simplified Anderson health service utilization model. We aim to (1) assess possible differences in utilization of health services between migrants and residents with “*Hukou*” in Beijing in the past month; (2) bring forth the major determinants influencing health seeking behaviors in different socioeconomic, demographic and health status setting. The comparison and inferences could help us to identify the barriers to using health services for migrants, and thus offer insight to targeted interventions to promote their health status.

### Analytic framework

On the basis of social systems approach, as a reliable tool, with decades of empirical support, Andersen health service utilization model is a well-validated theoretical framework aimed at understanding determinants of health services utilization, which takes into account both societal and individual determinants from the perspective of systematic analysis [19]. According to the model, health services utilization (including outpatient care, inpatient care, etc.) is determined by three dynamics: predisposing, enabling, and need variances (PEN). Predisposing factors can be social demographic characteristics such as age, race, sex, status which increase one’s need for health services. For instance, an individual who believes health service is an effective treatment for an ailment is more likely to seek care. Examples of enabling factors could be family support, access to health insurance, one’s community, etc. Enabling factors facilitate or impede use of health service. Need for care represents both self-perceived and actual need for health service.

**Fig. 1:**
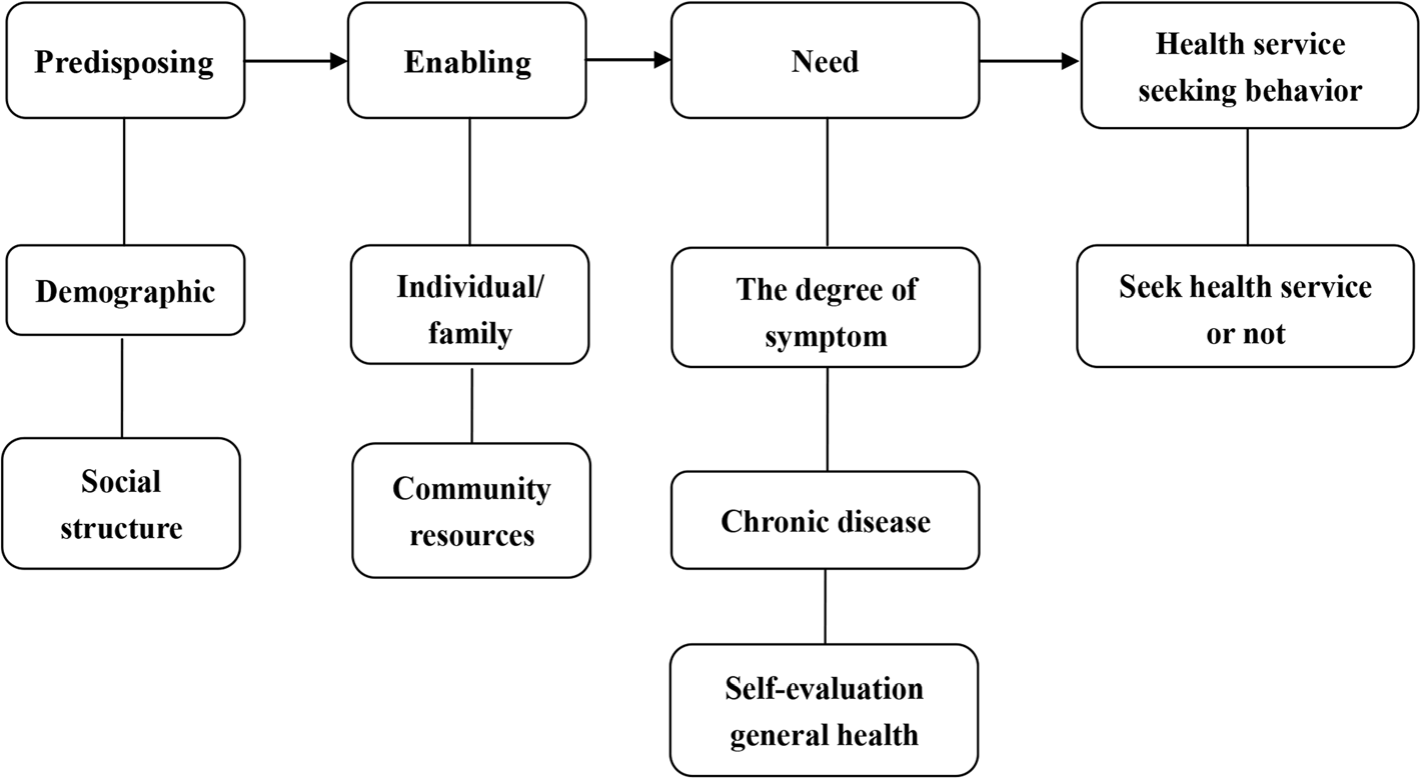
The simplified Anderson health service utilization model. Health service seeking behaviors (seek health service or not) is determined by three dynamics: predisposing (demographic and social structure), enabling (individual, family and community resources), and need variances (the degree of symptom, chronic disease and self-evaluation general health)

This model is further differentiated from its predecessors by using a feedback loop to illustrate that health outcomes may affect aspects such as needs (See Fig. 1). Our outcome variables was the utilization (seek health services, and non seek health services) of health services in the past month. We hypothesize that the PEN variables will be significant predictors of health services use, and that need variables will be the strongest predictors.

## Methods

### Ethics statement

The study was undertaken as a part of the National High Technology Research and Development Program-863 of the Chinese Government, which is a population-based cross sectional survey on risk factors of chronic non-communicable diseases (NCD). It was approved by the Ethical Committee of Capital Medical University, Beijing, China. Data were obtained from a 2012 cross-sectional survey in urban Beijing. Written informed consent was obtained from each participant involved in this study. For participants under the age of eighteen, written informed consents were obtained from their guardians. All participants’ information was kept confidential and tracked anonymously with identification number only.

### Data acquisition and study population

Data were collected from the survey of 2012-Beijing Urban Population Health Care Service Utilization. All participants were 15 years old and over, including both urban residents with Beijing resident certificate “*Hukou*” and inter-provincial migrants who lived in the sampling area for no less than 6 months, visitors to Beijing and those mentally unfit to respond were excluded. Considering decisions about medical care are customarily made by their parents or guardians, those under 15 years of age were not included in this study.

**Fig. 2:**
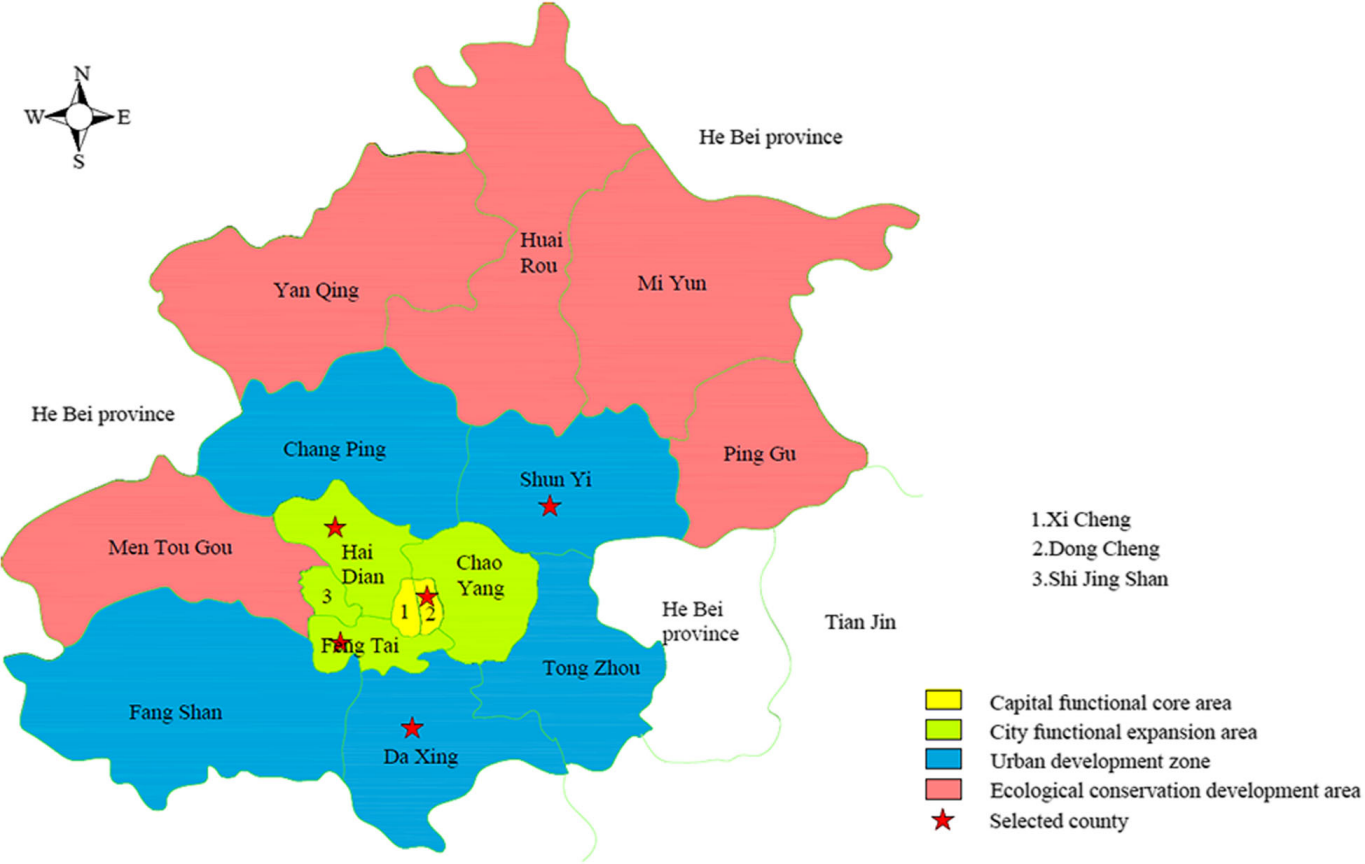
The map of Beijing (Reproduced by our research group). 16 counties are classified into four areas according to the functions in Beijing

The questionnaire was designed according to the contents of the Sixth China National Health Service Survey, and divided into four parts of contents as following: predisposing factors (demography and social structure), enabling factors (individual/family resources and community resources), need factors (general health condition), and health-seeking behavior (e.g., do nothing, self-treatment, and go to hospital). Fieldwork was performed from March to May, 2012. The quality assurance measures for this investigation included evaluating the questionnaire, training the investigators, and asking a fieldwork supervisor to monitor the investigation process. Before the investigation was implemented, it was reviewed, edited, and validated by experts from health administrative department, clinical teaching hospital and community health service institutions (CHSIs). The completed questionnaires, and validity of the collected data were independently checked. A pilot survey covering 400 persons was conducted between February 26 to 29, 2012 to assess applicability of the questionnaire and the fieldwork procedures. Beijing urban residents dwell mainly in three out of four areas were classified according to the functions of the county namely capital function core area (*Dongcheng* and *Xicheng* distircts), city functional expansion area (*Chaoyang*, *Haidian*, *Fengtai* and *Shijingshan* districts), urban development zone (*Tongzhou*, *Shunyi*, *Daxing*, *Fangshan* and *Changping* districts) and ecological conservation development area (*Mentougou*, *Pinggu*, *Miyun*, *Yanqing* and *Huairou* districts). A sample of 7000 adults was chosen from 5 of 16 counties in Beijing by using three-stage stratified and cluster random sampling. In the first stage, one county was selected from the capital functional core area (*Dongcheng* district), two counties were selected from the city functional expansion area (*Haidian* and *Fengtai* districts), and another two counties were selected from the urban development zone (*Daxing* and *Shunyi* districts) (See Fig. 2). In the second stage, two streets according to the economic condition and population size from each sampled county were selected. Finally, for each street selected, the total number of 7000 adults was recruited and investigated. The population was sampled in *Dongcheng* (1000), *Haidian* (2000), *Fengtai* (2000), *Daxing* (1000) and *Shunyi* (1000) respectively. All respondents were approached for face to face interview. Finally, 6592 out of the 7000 respondents were analyzed after excluding those whose information was missing on any of the study variables in the study. All data adopted double entry by using EpiData software (Version 3.1, EpiData association, Odense, Denmark). The two databases were compared and analyzed for discrepancies. If discrepancies exist, the original data source would be analyzed**.**

### Statistical analysis

A person-month was the unit of indicated receipt of services in a health setting at least once in a month, not the total number of times care received. Associations between categorical variables were tested using chi-square independence and binary logistic regression analysis. First, for the dataset, descriptive statistics and chi-square independence test stratified by nationality were calculated. Second, the binary logistic regression was performed in full model (considering all of the predisposing characteristics, enabling resources and the need factors were entered into the model, resulting in model I for migrants and model II for residents with “*Hukou*”), to estimate the potential major determinants that affect the patterns of health services utilization among two groups population, controlling for possible confounding associations on health services utilization. The dependent variable has two categories, including seek health services and non seek health services (self-medication or do nothing). The list of independent variables is summarized in the Table 1. All analyses were conducted using the IBM Statistical Package for Social Science software program (IBM-SPSS) Version 20.0 for Windows and all the tests are two sided. *P* < 0.05 was considered statistically significant, and the results were expressed as odds ratios (OR) with 95% confidence intervals (CI).

**Table 1:** The list of variables for empirical analysis

Predisposing	Demography	Age		15~ 24 (Reference group); 25~ 34;35~ 44;45~ 54;> 55
		Gender		Male (Reference group); Female
	Social structure	Marital status		Unmarried (Reference group); married; divorced/widowed
		Education level		Junior high school degree and below (Reference group); High school or secondary; University or college; Master’ degree or above
		Ethnicity		Han ethnic (Reference); Minorities
Enabling	Individual/family resources	Incoming monthly		< 3000 (Reference group); 3000~ 4999;> 5000
		Employment status		Formal work (Reference group); Retirement; Informal work
		Insurance status		Uninsured (Reference group); Insured
		Housing condition	Housing source	Own house (Reference group); Rent
	Community resources	Residential condition	Distance from residence to nearest medical institution	< 1 km (Reference group):1~;> 2
Need	Having chronic disease			No (Reference); Yes
	Having symptoms in the past month	Prevalence		No (Reference); Yes
		Severity		Mild (Reference); Moderate; Severe
	Self-evaluation general health status			Good (Reference); General; Poor

## Results

A total of 6592 Beijing urban residents above 15 years old were investigated, comprising 4578 residents (males 1654; females 2924) with “*Hukou*” and 2014 inter-provincial migrants (males 719; females 1295). Applying the population weights for both subgroups, respectively, the total sample represented 13,942,393 persons of the local urban population residents in Beijing in 2010 [4].

### Utilization of health services in the past month

The study revealed that 503 migrants (25.0%) and 1441 (31.5%) residents with “*Hukou*” reported at least one episode of discomfort in the past month. In the group of 25 to 34 years old, the rate of having symptoms of migrants was lower than that of residents with “*Hukou*” in the past month, but difference was not identified in other age groups (See Table 2).

**Table 2:** The health status of respondents in different age group in the past month

Age	Migrants (N, %)			Residents with “*Hukou*” (N, %)			*P-*value
	Total	Have one or more symptoms	%	Total	Have one or more symptoms	%	
15~	396	96	24.2	456	115	25.2	0.125
25~	859	179	20.8	825	217	26.3	0.005
35~	405	110	27.2	858	231	26.9	0.234
45~	197	50	25.4	960	295	30.7	0.076
55~	157	68	43.3	1479	583	39.4	0.098
Total	2014	504	25.0	4578	1442	31.5	< 0.0001

Table 3 indicates the differences of therapy measures selected between two groups population when they have symptoms in the past month. The rate of seeking health services one or more times was 46.7% for migrants as compared to 62.7% for residents with “*Hukou*”. The proportion of taking self-medication as well as taking nothing therapy measures were higher in migrants (24.3 and 29.0%) than residents with “*Hukou*” (15.2 and 22.1%).

**Table 3:** The therapy measures selected by respondents when they have symptoms in the past month

	Migrants		Residents with “*Hukou*”		*P-*value
	NO.	%	NO.	%	
Therapy measures					< 0.0001
Seek health services	235	46.7	903	62.7	
Self-medication	122	24.3	219	15.2	
Do nothing	146	29.0	319	22.1	

Descriptive statistics and chi-square independence test were used to analyze the difference in two groups of population by socio-economic and demographic factors. Chi-square independence test showed that age, ethnicity, employment status, having chronic disease and the degree of symptom were the major determinants affecting migrants to receive health services. For the residents with “*Hukou*”, the main influence factors included gender, age, ethnicity, marital status, employment status, education level, incoming, having chronic disease, self-evaluation of health and the degree of symptom in the past month (See Table 4).

**Table 4:** Information on the health utilization for persons who have symptoms by different characteristic in the past month

	Migrants (*n* = 503)					Residents with “*Hukou*” (*n* = 1441)				
Variances	Seek health services N (%)	Non seek health services N (%)	Total N (%)	χ2	*P*	Seek health services N (%)	Non seek health services N (%)	Total N (%)	χ2	*P*
Predisposing variables										
Gender				0.017	0.927				6.659	0.011
Male	89 (47.1)	100 (52.9)	189 (100)			276 (58.0)	200 (42.0)	476 (100)		
Female	146 (46.5)	168 (53.5)	314 (100)			627 (65.0)	338 (35.0)	965 (100)		
Age				14.937	0.005				69.937	0.000
15~	40 (41.7)	56 (58.3)	96 (100)			56 (48.7)	59 (51.3)	115 (100)		
25~	78 (43.6)	101(56.4)	179 (100)			107 (49.3)	110 (50.7)	217 (100)		
35~	46 (41.8)	64 (58.2)	110 (100)			126 (54.5)	105 (44.5)	231 (100)		
45~	25 (50.0)	25 (50.0)	50 (100)			178 (60.3)	117 (39.7)	295 (100)		
55~	46 (67.6)	22 (32.4)	68 (100)			436 (74.8)	147 (25.2)	583 (100)		
Ethnicity				11.001	0.001				0.007	1.000
Han	226 (48.9)	236 (51.1)	462 (100)			850 (62.6)	507 (37.4)	1357 (100)		
Minority	9 (22.0)	32 (78.0)	41 (100)			53 (63.1)	31 (36.9)	84 (100)		
Marital status				3.347	0.188				24.090	0.000
No married	70 (41.8)	100 (58.2)	170 (100)			96 (51.6)	90 (48.4)	186 (100)		
Married	156 (49.8)	157 (50.2)	313 (100)			711 (62.7)	423 (37.3)	1134 (100)		
Divorced/Widowed	9 (45.0)	11 (55.0)	20 (100)			96 (79.3)	25 (20.7)	121 (100)		
Education level				0.833	0.842				22.931	0.000
Junior high school and below	56 (50.0)	56 (50.0)	112 (100)			263 (71.9)	103 (28.1)	366 (100)		
High school or secondary	66 (46.5)	76 (53.5)	142 (100)			291(62.3)	176(37.7)	467(100)		
University or college	96 (44.9)	118 (55.1)	214 (100)			292 (58.9)	204 (41.1)	496 (100)		
Master’ degree or above	17 (48.6)	18 (51.4)	35 (100)			57 (50.9)	55 (49.1)	112 (100)		
Enabling variables										
Incoming				5.753	0.056				10.931	0.004
< 3000	135 (48.7)	142 (51.3)	277 (100)			589 (65.3)	313 (34.7)	902 (100)		
3000–4999	61 (39.4)	94 (60.6)	155 (100)			220 (61.1)	140 (38.9)	360 (100)		
> 5000	39 (54.9)	32 (45.1)	71 (100)			94 (52.5)	85 (47.5)	179 (100)		
Housing source				0.806	0.421				0.872	0.352
Own house	68 (50.0)	68 (50.0)	136 (100)			619 (63.5)	356 (36.5)	975 (100)		
Rent	167 (45.5)	200 (54.5)	367 (100)			284 (60.9)	182 (39.1)	466 (100)		
Employment status				6.365	0.041				46.566	0.000
Formal work	127 (48.8)	133 (51.2)	260 (100)			410 (59.7)	277 (40.3)	687 (100)		
Retirement	26 (60.5)	17 (39.5)	43 (100)			328 (75.1)	109 (24.9)	437 (100)		
Informal work	82 (41.0)	118 (59.0)	200 (100)			165 (52.1)	152 (47.9)	317 (100)		
Insurance				0.820	0.199				1.691	0.199
Uninsured	108 (44.6)	134(55.4)	242(100)			68 (57.1)	51 (42.9)	119 (100)		
Insured	127 (48.7)	134(51.3)	261(100)			835 (63.2)	487 (36.8)	1322 (100)		
Distance from residence to nearest medical institution				2.229	0.328				5.099	0.078
0~	85 (46.2)	99 (53.8)	184 (100)			391 (66.0)	201 (34.0)	592 (100)		
1~	31 (39.7)	47 (60.3)	78 (100)			182 (61.3)	115 (38.7)	297 (100)		
2~	119 (49.4)	122 (50.6)	241 (100)			330 (59.8)	222 (40.2)	552 (100)		
Need variables										
Having chronic disease				7.625	0.006				72.114	0.000
No	125 (41.7)	175 (65.3)	300 (100)			248 (48.2)	267 (51.8)	515 (100)		
Yes	110 (54.2)	93 (45.8)	203 (100)			655 (70.7)	271 (29.3)	926 (100)		
Self-evaluation general health status				4.840	0.089				8.213	0.016
Good	82 (42.3)	112 (57.7)	194 (100)			275 (62.4)	166 (37.6)	441 (100)		
Moderate	126 (47.7)	138 (52.3)	264 (100)			475 (60.5)	310 (39.5)	785 (100)		
Poor	27 (60.0)	18 (40.0)	45 (100)			153 (71.2)	62 (28.8)	215 (100)		
The degree of symptom in the past month				22.586	0.000				93.001	0.000
Mild	72 (36.7)	124 (63.3)	196 (100)			199 (46.9)	225 (53.1)	424 (100)		
Moderate	127 (49.2)	131 (50.8)	258 (100)			527 (65.1)	283 (34.9)	810 (100)		
Severe	36 (73.5)	13 (26.5)	49 (100)			177 (85.5)	30 (14.5)	207 (100)		

### Logistic regression model

The finding reveals that the full models for migrants and residents with “*Hukou*” containing all predictors were statistically significant, (model I, χ2 = 41.059, *p* = 0.000; model II, χ2 = 190.112, *p* = 0.000), indicating that the models was able to distinguish between respondents who seek health services and those who do not to seek health services after their having symptoms.

In the model summary, the model I as a whole explained between 7.8% (Cox and Snell R square) and 10.5% (Nagelkerke R square) of the variance in health services utilization of migrants was explained by all the predictor variables entered into the logistic regression model. The model II as a whole explained between 12.4% (Cox and Snell R square) and 16.9% (Nagelkerke R square) of the variance in health services utilization of residents with “*Hukou*” was explained by all the predictor variables entered into the logistic regression model (See Table 5).

**Table 5:** Model summary of health services utilization in the past month for two groups population

	-2Log likelihood	Cox and Snell R Square	Nagelkerke R Square
Model I^a^	654.081	0.078	0.105
Model II^b^	1714.070	0.124	0.169

Model I: Binary logistic regression analysis of predictors of health services utilization of migrants in the past month

Model II: Binary logistic regression analysis of predictors of health services utilization of the residents with “*Hukou*” in the past month

^a^χ^2^ = 41.059, *P* = 0.000

^b^χ^2^ = 190.112, P = 0.000

Tables 6 and 7 predict the determinants of health services utilization for migrants (model I) and residents with “*Hukou*” (Model II) by binary logistic regression. ModelIin Table 4 shows the migrants with moderate degree and severe degree of symptom in the past month were at 1.623-times (OR = 1.623) and 5.035-times (OR = 5.035) higher chances of seeking health services respectively, comparing to the mild degree of symptom. Minority migrants were less likely to seek health services than Han migrants (OR = 0.282, 95%-CI 0.128, 0.621, *P* < =0.002). Model II in Table 5 indicates that for the residents with “*Hukou*”, female (OR = 1.314), age above 55 years old (OR = 2.253), having chronic disease (OR = 1849), with moderate and severe degree of symptom in the past month (OR = 2.032 and OR = 5.786), were more likely to seek health services. Additionally, the chances of seeking health services were decreased by 33.3% in moderate health status (OR = 0.667) and 38.2% in poor health status (OR = 0.618), comparing to self-evaluated good health status.

**Table 6:** Binary logistic regression analysis of predictors of health services utilization of migrants in the past month

Variables in the Equation	Model I			
	B (SE)	Wald	OR [95%-CI]	*P*-value
Predisposing variables				
Minorities **(**Ref = Han ethnic**)**	−1.265 (0.403)	9.871	0.282 [0.128, 0.621]	0.002
Enabling variables				
Incoming **(**Ref = < 3000)				
3000~ 4999	−0.400 (0.211)	3.590	0.670 [0.443,1.014]	0.058
> 5000	0.322 (0.276)	0.276	1.380 [0.804,2.370]	0.243
Need variables				
The degree of the symptom in the past month (Ref = Mild)				
Moderate	0.484 (0.197)	6.029	1.623 [1.103, 2.389]	0.014
Severe	1.616 (0.364)	19.739	5.035 [2.468, 10.272]	0.000
Constant	−0.368 (0.176)	4.349	0.615	0.058

*Abbreviation*: *B*: Unstandardized regression coefficient, *SE* Standard error, *OR* Odds ratio, *CI* Confidence interval, *Ref* Reference category

Model I: Binary logistic regression analysis of predictors of health services utilization of migrants in the past month

**Table 7:** Binary logistic regression analysis of predictors of health services utilization of the residents with “*Hukou*” in the past month

Variables in the equation	Model II			
	B (SE)	Wald	OR [95%-CI]	*P*-value
Predisposing variables				
Female (Ref = Male)	0.273 (0.124)	4.859	1.314 [1.031, 1.674]	0.028
Age (Ref = 15~ 24)				
25**~** 34	0.287 (0.319)	0.809	1.333 [0.713, 2.491]	0.368
35**~** 44	0.548 (0.371)	2.178	1.730 [0.835, 3.581]	0.140
45**~** 54	0.327 (0.372)	0.773	1.387 [0.669,2.873]	0.379
> 55	0.812 (0.381)	4.556	2.253 [1.069, 4.751]	0.033
Marital status (Ref = Single)				
Married	−0.492 (0.285)	2.976	0.611 [0.349, 1.069]	0.085
Divorced/Widowed	0.034 (0.375)	0.008	1.035 [0.496, 2.158]	0.928
Employment status (Ref = Formal work)				
Retirement	0.229 (0.169)	1.827	1.257 [0.902, 1.752]	0.177
Informal work	−0.241 (0.153)	2.469	0.786 [0.582, 1.061]	0.116
Need variables				
Having chronic disease (Ref = No)	0.615 (0.145)	18.060	1.849 [1.393, 2.455]	0.000
The degree of the symptom in the past month (Ref = Mild)				
Moderate	0.709 (0.130)	29.959	2.032 [1.576, 2.619]	0.000
Severe	1.755 (0.236)	55.430	5.786 [3.645, 9.184]	0.000
Self-evaluation general health status (Ref = Good)				
Moderate	−0.405 (0.135)	9.095	0.667 [0.512, 0.868]	0.003
Poor	−0.481 (0.207)	5.409	0.618 [0.412, 0.927]	0.020
Constant	−0.486 (0.256)	3.602	0.692	0.037

*Abbreviation*: *B*: Unstandardized regression coefficient, *SE* Standard error, *OR* Odds ratio, *CI* Confidence interval, *Ref* Reference category

Model II: Binary logistic regression analysis of predictors of health services utilization of the residents with “*Hukou*” in the past month

## Discussion

This study attempted to describe the health status and health services behaviors of migrants, and assess the major determinants associated with the health services utilization in an urban setting in Beijing by Anderson health service utilization model, and to better facilitate health services utilization for migrants.

### Health status

Consistent with the theory of “healthy migrant effect” that first-generation migrants are often healthier with lower overall morbidity and mortality than local-born populations [20], our study also find that the recent one-month prevalence rate of illness among migrants was lower than residents with “*Hukou*” without classified by age group. Subsequently, a large number of studies find that the migrant health advantage diminishes dramatically especially in middle age [21, 22]. In our study, migrants had lower prevalence rate of illness than that of residents with “*Hukou*” in the group of 25 to 34 years old, but the distinction was not revealed in other age groups. Meanwhile, the recent one-month physician visit rate in the past month among migrants was lower than residents with “*Hukou*”. It remains a concern that migrants are often over-optimistic about their health status due to the poor understanding of health which is induced by the socioeconomic environment they live and work in [23].

### Influence factors of health services utilization

In the study, the finding validated our hypothesis that need factors (having chronic disease, self-evaluation general health status, and the degree of symptom) were the most dominant predictors of health services utilization especially for residents with “*Hukou*”, even when adjusting for predisposing and enabling variables. Migrants in the moderate and severe status of symptom have higher likelihood of seeking healthcare services. There might be some factors account for the differences. Firstly, these results might be due to the fact that the migrants are relatively young, i.e. nearly half of respondents were aged between 15 and 35 in this and other studies [8, 24]. Selection bias in migration indicated that those who voluntarily migrate tend to have healthier lifestyles and lower prevalence rate of illness than either their urban counterparts born in host countries or their non-migrating peers in the original places [20]. Secondly, under the pressures of heavy working, younger migrants always have a satisfactory level of self-reported health risk awareness, and were more inclined to ignore the health problems than their urban counterparts [11]. Furthermore, considering high health expenses, along with deficiency in health insurance for lowering health risks, they might be more likely to take regular medication or palliative care instead of seeking healthcare services in medical institutions when they only have minor illness. Finally, most of migrants would return to their hometown to treat severe illness or enjoy old age, from an economical perspective [25]. For the above reasons, most migrants made use of health services only when they were in serious health status.

For migrants, we observed that ethnicity of predisposing variables contributed significantly to the variance in health services utilization. Ethnic minority migrants are less likely to use health services than Han migrants, and the result was consistent with the previous research of Guangxi [26]. The possible explanations could be divided into two aspects, one is that ethnic minority migrants mainly rely on relatives, friends, or fellow villagers to acquire resources in urban communities. Social assistance networks were rarely provided by organizations, such as grassroots government, community neighborhood committees, enterprises, civil organizations [27]. On the other hand, special religious beliefs might affect their health service seeking behaviors. They might pray to god or spirit for relieving their sufferings, or take special herb medicine to cure or alleviate their illness instead of seeking health services [28]. Taking into account the sample size of minority selected is small in this study, in the future, ethnic minority migrants should be studied in terms of their health service utilization behaviors and influencing factors as a unique group.

In this research, the result was different from some previous China-based studies which indicated that insurance participation increases the likelihood of seeking health services in the general population [29]. Comparing to residents with “*Hukou*”, less migrants had insurance in Beijing. The root cause of this phenomenon is the household registration system (*Hukou*) in China, which divides the population into rural and urban citizens [30]. Although the “*Hukou*” system is no longer used to prevent the flow of migrants, the obstacles for the majority of interprovincial migrants acquired permanent urban residency rights and many of the associated social welfare still existed. China’s government launched the policy of “New Rural Cooperative Medical System” (NRCMS) for rural “*Hukou*” residents to provide basic healthcare in 2003. By the end of 2013, 98.7% (802 million) of the rural population were covered by the NRCMS. There is no doubt that NRCMS improved both hospitalization and outpatient services utilization to prevent catastrophic health payment in rural China, yet it was useless for migrants with rural “*Hukou*” which account for 80% of the total migrants in immigration cities [31, 32]. Many migrants had insurance in their hometown, and it is nearly impossible to transfer insurance relations between different provinces in China. Thus, they need to return to hometown to reimburse their medical expense. Although “migrant worker insurance” was launch in 2006, it covered the migrant workers with stable employment relation with urban employers, and was not applicable for the self-employed or the unregistered migrants [33]. Working without a contract is common to labor migrants in most industries in majority immigrant countries, which often can prevent privilege to the typical national or local health insurance, support services or social assistance [34–36]. Lower health insurance coverage and complicated reimbursement procedure increased the barriers to access health services for migrants. For the reasons mentioned above, health insurance is not a determinant to health services utilization for migrants.

#### Limitation

Our study is one of the first to apply Andersen’s health service utilization model to analyze potential determinants of health service utilization of migrant and residents with “*Hukou*” in Beijing. The study has several limitations. Firstly, our study uesd a cross-sectional design, and causality cannot be determined. Secondly, the information collected was self-reported in the past month. Recall biases might induce the underreported information of health status and health utilization. Thirdly, health utilization in the past month was measured as a dichotomous variable, and the variables examined may more logically account for the use or non-use of health services rather than measured in terms of health services visit intensity. Finally, some important potential factors may have been neglected in the model, such as personal behaviors or biological risk factors. Future research is needed to identify more variables to explain the causal relationships among the variables of Anderson’s health service utilization model.

## Conclusion

This survey contributes to our understanding of the health status and health seeking behavior among Chinese migrants and residents with “*Hukou*” in the past month. Compared with the residents with “*Hukou*”, migrants had better health status in the group of 25 to 34 years old, and lower rate of health services utilization. In particular, the moderate and severe status of symptom in the past month, representing the need factors in the Anderson health service utilization model, was a dominant predictor of health services utilization for migrants. Additionally, ethnicity of predisposing variables contributed significantly to the variation in health services utilization. In the future, we should pay special attention to the health services utilization for minority migrants. Although migrants are marginalized group, every person in a civil society has the right to access adequate health care. The results of this study would be useful for highlighting health care system reform. It suggests that policy makers should take measures to break down barriers for migrants, including establishment of policies and processes that ensure easy and equitable access to health care, such as increasing the coverage of health insurance, and implementing culturally appropriate promotion of health habits and behaviors. In the future, more comprehensive researches are needed to study the extent of the gap of health utilization between migrants and local-born populations to improve the access and utilization of health services for migrants.

## Abbreviations

CHSIs: Community health service institutions
MHRSS: Ministry of Human Resources and Social Security
NCD: Chronic non-communicable diseases
NRCMS: New Rural Cooperative Medical System
